# Portraits of New-York Medical Professors

**Published:** 1846-08

**Authors:** 


*Portraits of New-York Medical Professors.* Messrs. S. & W. Wood, No, 261 Pearl street, New-York, have an immense collection of works on medicine, anatomy, surgery, midwifery, chemistry, &c., which are worthy the examination of medical gentlemen visiting that city. In their recently-published catalogue, we notice that they have the portraits of the faculty of the two medical colleges of New-York. The seven professors of each institution are on separate plates, of twenty-four by thirty inches. A few of the impressions would be regarded with satisfaction this way. There is a peculiar gratification in knowing how those men look, who have notified the world by their intellectual efforts, that they are active and useful in it. —*Boston Med. and Surg. Journal.*

